# Applications of biophysical techniques in drug discovery and development

**DOI:** 10.5599/admet.767

**Published:** 2019-12-11

**Authors:** Tatjana Ž. Verbić

**Affiliations:** University of Belgrade – Faculty of Chemistry, Studentski trg 12-16, 11000 Belgrade, Serbia

Drug discovery and development in many pharmaceutical companies and academic laboratories have benefited from the rapid advancements in biophysical experimental methods over the last two decades. At the end of the 20^th^ century, application of these methods was still restricted to a (relatively) small number of scientists using specialized, low throughput technologies and methods. Now, automated high-throughput instruments are to be found in a growing number of laboratories [1]. Techniques such as mass spectrometry, multidimensional nuclear magnetic resonance spectroscopy, image processing, X-ray diffraction, electron microscopy, atomic force microscopy, fluorescence spectroscopy, dynamic light scattering, surface plasmon resonance spectroscopy, differential scanning calorimetry and isothermal titration calorimetry are now routinely used in many key stages of the drug discovery and development.

Generally speaking, biophysical methods are in two ways involved in drug design: the qualitative detection of small molecule binding to a target (hit identification), and the quantitative determination of physical parameters associated to binding (hit-to-lead progression) [2]. In the first case, efforts have been made toward miniaturization, automation, and speed-up of the screening process allowing higher throughput. In the second one, sophisticated applications have been developed to derive detailed relevant information.

The demand for more automated and higher-throughput versions of instrumentation continues to grow. With such advancement in instrumentation, great improvements in the speed, sensitivity and the range of possible biophysical measurements have been reached. High-resolution mechanistic, kinetic, thermodynamic and structural information on drug-target interactions are now available. In particular, biophysical measurements are useful in supporting compound progression, mechanistic understanding of the drug-receptor binding, validating potency data from biochemical and cellular assays in the discovery phases and quality control of the investigational drug, including the evaluations of drug release and stability, in the development phases.

In view of the rapid advancement and the important roles of biophysical techniques in drug discovery and development, *ADMET and DMPK* devoted a special issue to this topic. Among received articles, three were chosen to be published, one original scientific article and two reviews. In the following issues of the journal a couple of more articles are expected to be published.

The manuscript written by G. Holdgate, K. Embrey et al. [3] provides a review about the use of biophysical methods in early phases of drug discovery, which represents an update of previously published book chapter [1]. The use of various biophysical techniques is discussed, including thorough assay development, primary screening, hit confirmation, and mechanistic characterization. Each method has its own advantages and disadvantages that lead to different applications dependent upon the reagents available and the information content desired. Two case studies are presented.

The use of NMR spectroscopy in the various drug discovery and development processes is discussed in the manuscript written by M. Zloh [4]. Applications of hydrogen nuclear magnetic spectroscopy (^1^H NMR spectroscopy), not often reported as a tool for evaluating some ADMET properties, are explored. The use of quantitative NMR (qNMR) in solubility, lipophilicity (log *P*) and acidity constants (p*K*_a_) determinations is discussed.

The work by I. Quiroga and T. Scior [5] describes the role of induced fit mechanism in binding to cytochrome P450 3A4. Root means square deviations (RMSD values) of ligand bound/unbound structures of 3A4 are analyzed after the molecular dynamic (MD) simulations. The study demonstrates that the smallest dynamic differences are met in pairs which share either the presence or the absence of bound substrates. Substrate alone does provide an effect on the geometric changes of the enzyme which can be observed and measured in MD studies.

The guest editor would like to thank all the contributors for the time and efforts put in presented manuscripts. It is hoped that readers will find these contributions useful in their research.
